# Electrochemical selection and characterization of a high current-generating *Shewanella oneidensis* mutant with altered cell-surface morphology and biofilm-related gene expression

**DOI:** 10.1186/1471-2180-14-190

**Published:** 2014-07-16

**Authors:** Atsushi Kouzuma, Hitomi Oba, Nozomi Tajima, Kazuhito Hashimoto, Kazuya Watanabe

**Affiliations:** 1 School of Life Sciences, Tokyo University of Pharmacy and Life Sciences, 1432-1 Horinouchi, Hachioji 192-0392, Tokyo, Japan; 2 Department of Applied Chemistry, The University of Tokyo, 7-3-1 Hongo, Bunkyo-ku 113-8656, Tokyo, Japan; 3 Hashimoto Light Energy Conversion Project, ERATO/JST, The University of Tokyo, 7-3-1 Hongo, Bunkyo-ku 113-8656, Tokyo, Japan; 4 Present address: Advanced Technologies Research Laboratories, Idemitsu Kosan, 1200 Kamiizumi, Sodegaura 299-0293, Chiba, Japan

**Keywords:** Extracellular electron transfer, Microbial fuel cell, Anode respiration, Bioelectrochemical systems

## Abstract

**Background:**

*Shewanella oneidensis* MR-1 exhibits extracellular electron transfer (EET) activity that is influenced by various cellular components, including outer-membrane cytochromes, cell-surface polysaccharides (CPS), and regulatory proteins. Here, a random transposon-insertion mutant library of *S. oneidensis* MR-1 was screened after extended cultivation in electrochemical cells (ECs) with a working electrode poised at +0.2 V (vs. Ag/AgCl) to isolate mutants that adapted to electrode-respiring conditions and identify as-yet-unknown EET-related factors.

**Results:**

Several mutants isolated from the enrichment culture exhibited rough morphology and extraordinarily large colonies on agar plates compared to wild-type MR-1. One of the isolated mutants, designated strain EC-2, produced 90% higher electric current than wild-type MR-1 in ECs and was found to have a transposon inserted in the *SO_1860* (*uvrY*) gene, which encodes a DNA-binding response regulator of the BarA/UvrY two-component regulatory system. However, an in-frame deletion mutant of *SO_1860* (∆SO_1860) did not exhibit a similar level of current generation as that of EC-2, suggesting that the enhanced current-generating capability of EC-2 was not simply due to the disruption of *SO_1860*. In both EC-2 and ∆SO_1860, the transcription of genes related to CPS synthesis was decreased compared to wild-type MR-1, suggesting that CPS negatively affects current generation. In addition, transcriptome analyses revealed that a number of genes, including those involved in biofilm formation, were differentially expressed in EC-2 compared to those in ∆SO_1860.

**Conclusions:**

The present results indicate that the altered expression of the genes related to CPS biosynthesis and biofilm formation is associated with the distinct morphotype and high current-generating capability of strain EC-2, suggesting an important role of these genes in determining the EET activity of *S. oneidensis*.

## Background

*Shewanella* species, which are affiliated with the class *Gammaproteobacteria*, are widely distributed in nature, including marine, freshwater, sedimentary, and soil environments [1]. Members of this genus are able to respire various organic and inorganic compounds (e.g., oxygen, fumarate, nitrate, nitrite, thiosulfate, and elemental sulfur), as well as soluble and solid metals (e.g., iron, manganese, uranium, chromium, cobalt, technetium, and vanadium) [2-5]. In recent years, a few *Shewanella* species have attracted considerable attention due to their potential applicability for bioremediation [6] and bioelectrochemical systems (BESs), such as microbial fuel cells (MFCs) and microbial electrosynthesis cells [7-10].

*Shewanella oneidensis* MR-1 is the most extensively studied strain in the genus *Shewanella* because of its metabolic versatility [11], annotated genome sequence [12,13], and ease of genetic manipulation [14]. In addition, since it was shown in 1999 that strain MR-1 has the ability to transfer electrons to an extracellular electrode without exogenously added mediator [7], it has served as a model bacterium for studying microbial current generation and extracellular electron transfer (EET) pathways [1,15]. These studies have revealed that MR-1 cells have multiple EET pathways, including direct EET pathways that involve outer-membrane cytochromes (OM-cyts) [15] and electrically conductive pilus-like structures (nanowires) [16,17], and indirect EET pathways that function via self-produced electron shuttle compounds, such as flavins [18-21].

Despite these studies, a deeper understanding of the molecular mechanisms of EET is required to optimize and enhance microbial electron transfer rates in BESs because studies in *S. oneidensis* MR-1 have also indicated that EET is a complex process that is influenced by various intracellular and extracellular components. Saffarini et al. [22] and Charania et al. [23] have revealed that cyclic AMP (cAMP) and cAMP receptor proteins are necessary for the expression of OM-cyts. In addition, Covington et al. [24] identified the *ushA* gene, which is involved in flavin secretion in MR-1. Our previous studies have also suggested the possibility that extracellular components, including cell-surface polysaccharides (CPSs), are involved in EET and current generation in BESs [25,26]. It is therefore conceivable that many unknown factors are also involved in EET.

A useful approach for identifying unknown cellular components (and genes) associated with a particular phenotype involves the construction and screening of random mutant libraries for mutants with altered phenotypes. We previously succeeded in isolating MR-1 mutants with increased current-generating activities from a transposon (Tn) insertion mutant library that was cultured under electrode-respiring conditions [25,26]. Notably, the obtained mutants had an altered (rough) colony morphology on agar plates [25,26], indicating that the electrochemical cultivation and subsequent screening on agar plates of random Tn insertion mutants is a useful approach for isolating current-generating mutants. Although our previous studies have identified several genes related to cell-surface morphology and current generation [25,26], it is reasonable to speculate that many other unknown factors remain to be identified, considering the complexity of bacterial cell-surface structures and EET processes.

In the present study, we screened MR-1 mutants from a random Tn insertion library for altered colony morphology on agar plates after selection in electrochemical cells (ECs), to identify mutants with enhanced current-generating capability. One of the isolated mutants, designated strain EC-2, formed extraordinary large colonies on agar plates and generated higher electric current in an EC than wild-type (WT) MR-1. Analyses of strain EC-2 revealed that a number of genes, including those involved in CPS synthesis and biofilm formation, were differentially expressed compared to WT, suggesting that these genes were associated with the distinct phenotype of this mutant.

## Results

### Electrochemcial selection of mutants

A library of *S. oneidensis* MR-1 random transposon mutants was introduced into an EC reactor equipped with a working electrode poised at +0.2 V (*vs.* Ag/AgCl) and cultivated under electrode-respiring conditions. A current versus time curve generated during the electrochemical cultivation of MR-1 is shown in Additional file 1: Figure S1. After 40-days of electrochemical cultivation, the electrolyte was sampled spread on agar plates for the isolation of mutant cells. We found that approximately 20% of colonies formed by the electrochemically cultivated mutants were larger than those of WT MR-1, suggesting that the mutants with large-colony morphology grew preferentially under the electrode-respiring conditions.

**Figure 1:**
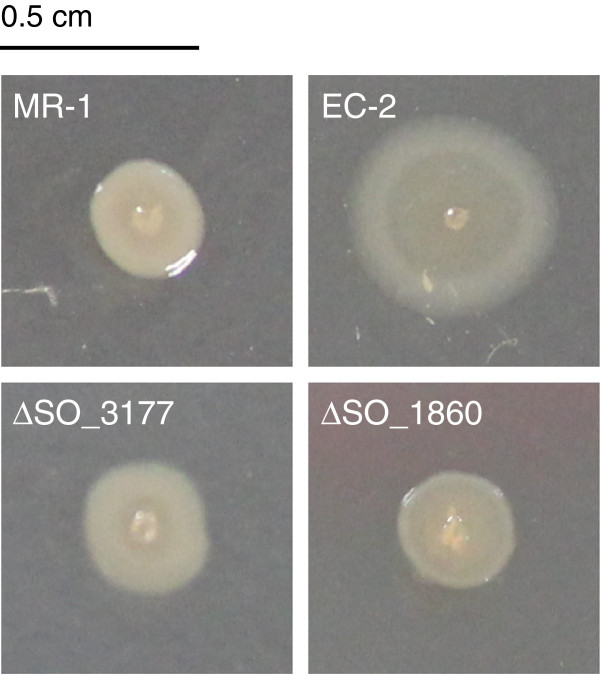
**Colony morphology of *S. oneidensis *** **MR-1 and mutant strains on LB agar plates.**

One of the isolated mutants, designated strain EC-2, was clearly distinct from the other mutants and WT strains as it formed an extraordinary large colony (Additional file 2: Figure S2 and Figure 1). We previously found that a CPS-deficient mutant of MR-1, ∆SO_3177, formed large, flat, and rough colonies on agar plates, and generated higher power output in MFCs compared to that of WT [25], indicating an association between cell-surface morphology and current generation. Strain EC-2 also exhibited a rough, flat colony morphology, but formed larger colonies than those of ∆SO_3177 (Figure 1). Strain EC-2 was therefore selected for further characterization, including genetic analyses, to determine the identity and function of the mutated gene(s) in this mutant.

### Evaluation of strain EC-2 in MFC

**Figure 2:**
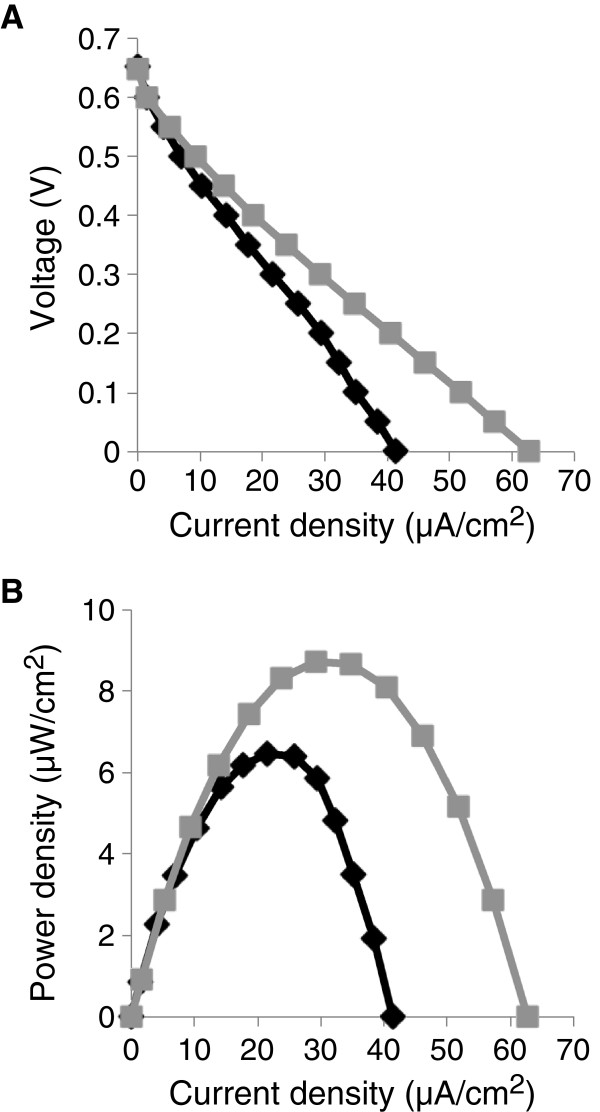
**Evaluation of strain EC-2 in MFCs. (A)** Polarization and **(B)** power curves for MFCs containing strain EC-2 (grey line) and wild-type (WT) MR-1 (black line) are shown.

The ability of strain EC-2 to generate electrical power in an MFC was analyzed and compared with that of WT (current versus time curves are shown in Additional file 3: Figure S3). To evaluate MFC performance, polarization (Figure 2A) and power (Figure 2B) curves were determined for each MFC after the current generation became stable (day 14 in Additional file 3: Figure S3). The *P*_max_ and *I*_sc_ for the EC-2 MFC (8.74 μWcm^-2^ and 62.7 μAcm^-2^, respectively) were approximately 1.5-fold higher than those for the WT MFC (6.46 μWcm^-2^ and 41.3 μAcm^-2^, respectively).

**Figure 3:**
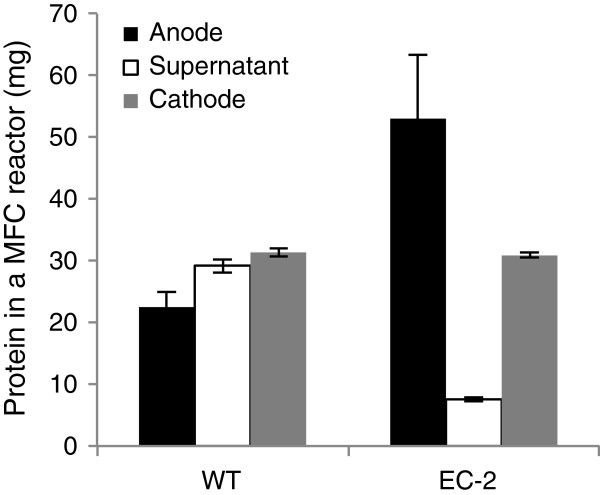
**Protein contents of the anode biofilms, supernatant, and cathode biofilms in single-chamber MFCs, showing the distribution of wild-type (WT) and EC-2 cells.** Error bars represent standard deviations of means that were calculated from at least four assays.

In the single-chamber MFC used in this study, *Shewanella* cells were localized to either the electrolyte as planktonic cells, or to the anode and cathode, where they formed biofilms. For strain EC-2, the turbidity of the MFC electrolyte was markedly lower than that in the WT MFC (data not shown), suggesting that more mutant cells were attached to the electrodes. To examine this hypothesis, WT and EC-2 cells from the electrolyte and electrodes of each MFC were collected after approximately 360 h of operation, and the protein contents in these samples were measured to estimate cell concentration (Figure 3). In the EC-2 MFC, the amount of cells attached to the graphite felt anode (53.0 ± 10.2 mg) was 140% higher than that of WT cells (22.5 ± 2.4 mg), whereas the amount of EC-2 cells in the electrolyte (7.6 ± 0.3 mg) was 74% lower than that of WT cells (29.2 ± 1.1 mg). The total protein contents, which represented the sums of the protein contents in the three MFC areas, did not significantly differ between the two strains. Together, these results indicate that strain EC-2 has the increased ability to adhere to the graphite felt anode when cultivated under electrode-respiring conditions.

### Identification of the Tn-insertion site in strain EC-2

PCR analysis of strain EC-2 revealed that a Tn was inserted into the *SO_1860* gene, whose deduced amino acid sequence showed a significant homology (70% identity) to the DNA-binding response regulator UvrY of *Escherichia coli*. In *E. coli*, UvrY is part of the BarA/UvrY two-component regulatory system [27], which regulates the Csr (carbon storage regulation) system via transcriptional activation of CsrB, a small noncoding RNA [28,29]. It has been reported that the BarA/UvrY/Csr regulatory cascade in *E. coli* is involved in the regulation of numerous physiological functions, including carbon metabolism, motility, adhesion, and biofilm formation [28,29]. Recent studies also revealed that the BarA/UvrY/Csr regulatory cascade is conserved in *S. oneidensis* MR-1, and that *SO_1860* (UvrY) is involved in the transcriptional regulation of more than 200 genes, including those involved in CPS biosynthesis genes [30] and biofilm formation [31]. However, the roles of *SO_1860* in EET and current generation have not yet been investigated.

### Deletion of the *SO_1860* gene

**Figure 4:**
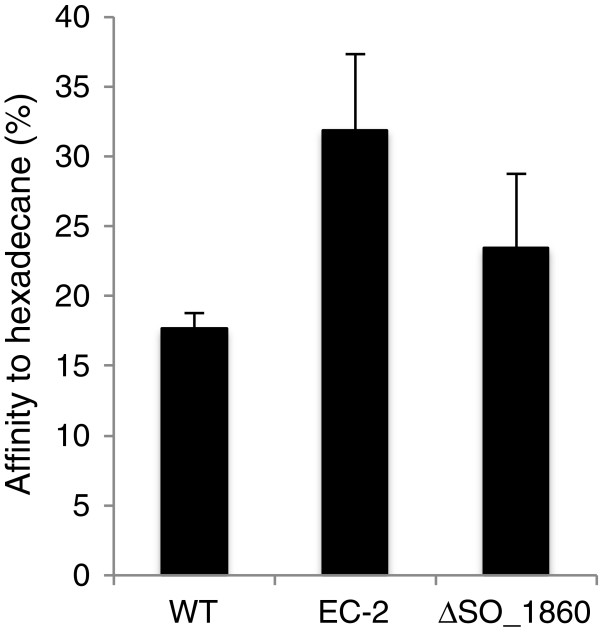
**Hydrophobic properties of WT, EC-2, and ∆SO_1860 cells.** Percentages of cells partitioned into the hydrophobic solvent hexadecane are shown. Error bars represent standard deviations of means that were calculated from at least 6 replicates. All calculated means differed significantly from each other (*P* < 0.05).

To confirm that disruption of the *SO_1860* gene was responsible for the distinct morphotype and enhanced current generation by strain EC-2, an in-frame deletion mutant of *SO_1860* (designated ∆SO_1860) was constructed. When cultivated on LB plates, ∆SO_1860 displayed a slightly rough colony morphotype as compared with WT (Figure 1). However, colonies formed by ∆SO_1860 were similar in size to those of WT, and smaller than those of strain EC-2. As colony morphology is influenced by cell surface structures and physicochemical properties [32-34], we next evaluated cell surface hydrophobicity of WT, EC-2, and ∆SO_1860 by measuring the affinity of cells to hexadecane (Figure 4). The EC-2 and ∆SO_1860 mutants were more hydrophobic than WT, but the hydrophobicity of ∆SO_1860 was lower than that of EC-2. These results were consistent with the rough colony appearance of both mutant strains.

**Figure 5:**
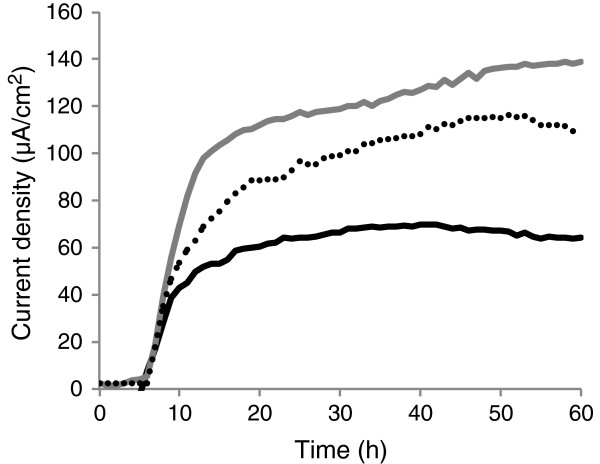
**Comparison of current generation by WT (black line), EC-2 (grey line), and ∆SO_1860 (dotted line) in ECs.** Results represent means of at least two parallel but independent experiments.

Current generation by ∆SO_1860 was also analyzed and compared with that of WT and strain EC-2 (Figure 5) using a small-volume, single-chamber EC reactor equipped with a working electrode poised at +0.2 V (*vs.* Ag/AgCl) for the stable and short-term measurement of electric current (Figure 5). Strain EC-2 and ∆SO_1860 generated 90% and 60%, respectively, higher current than WT. These results indicated that although the disruption of *SO_1860* affected colony morphology, cell surface hydrophobicity, and current-generating ability, it was not the only cause for the distinct phenotype of strain EC-2. The reason for this will be discussed later.

### Expression of CPS biosynthesis genes

**Figure 6:**
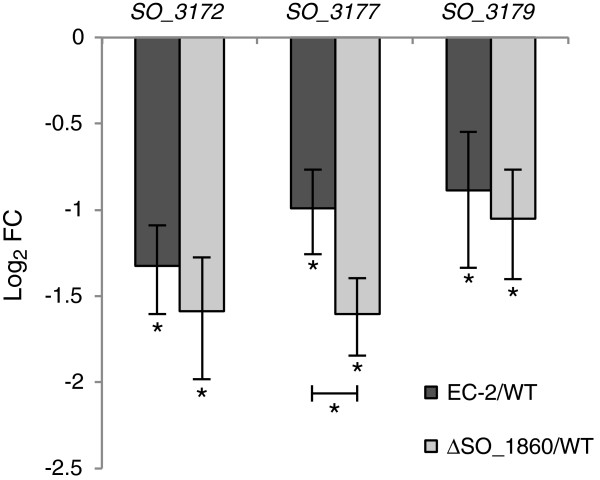
**qRT-PCR analyses of CPS biosynthesis genes in WT, EC-2, and ∆SO_1860 cells.** Results are expressed as log_2_-fold changes (FC) relative to expression levels in WT cells. The error bars represent the standard deviation calculated from at least four measurements. Asterisks indicate a statistically significant difference (*P* < 0.05) between expression levels in the three strains.

Our previous study found that CPSs negatively affect current generation in *S. oneidensis* MR-1 [25]. In addition, the CPS biosynthesis genes, which are located within a large gene cluster (*SO_3193* to *SO_3171*), are reported to be down-regulated in a *SO_1860* knockout mutant [30]. We therefore hypothesized that CPS biosynthesis was repressed in strain EC-2, resulting in the increased ability of this mutant to generate current. To examine this hypothesis, WT, EC-2 and ∆SO_1860 cells were grown in LMM under fumarate-reducing conditions, and expression levels of three CPS biosynthesis genes (*SO_3172*, *SO_3177*, and *SO_3179*) were determined by the quantitative reverse transcription PCR (qRT-PCR) analysis of extracted total RNA (Figure 6). In both EC-2 and ∆SO_1860, expression levels of the CPS biosynthesis genes were decreased to 40%–54% of those in WT (log_2_-transformed fold changes [log_2_ FC] < -0.89), supporting the speculation that the repression of CPS is related to the increased current generation by these two mutant strains. However, the expression levels of *SO_3172* and *SO_3179* did not significantly differ between EC-2 and ∆SO_1860, and the expression level of *SO_3177* was slightly decreased in ∆SO_1860 as compared with that in EC-2. These results suggest that the high current-generating capability of EC-2, which exceeded that of ∆SO_1860, was not simply due to the decreased expression of CPS biosynthesis genes.

### Differentially expressed genes in EC-2

To investigate why EC-2 exhibited a distinct phenotype from ∆SO_1860, gene expression profiles in the two mutants were analyzed and compared by transcriptome analysis. Total RNA from EC-2 and ∆SO_1860 was prepared from cells cultured in LMM under fumarate-reducing conditions and subjected to microarray analysis. The reliability of the microarray analysis was validated by qRT-PCR of five selected genes (Additional file 4: Figure S4). A high correlation (*r*^2^ = 0.97) was observed between the microarray and qRT-PCR results.

Microarray analysis revealed that 26 genes had significantly different expression levels (*P* < 0.05, |log_2_ FC| ≥ 1.0) in strains EC-2 and ∆SO_1860 (Table 1). Among the 26 genes, 11 genes were up-regulated and 15 genes were down-regulated in strain EC-2 as compared with ∆SO_1860. The up-regulated genes included those assigned to the COG (Clusters of Orthologous Groups of proteins [35]) categories of “Transcription”, “Signal transduction mechanisms”, “Nucleotide transport and metabolism”, and “Amino acid transport and metabolism”. Notably, expression levels of the genes involved in methionine biosynthesis, *metR* and *metE*, were remarkably up-regulated in EC-2. It is known that MetR acts as a transcriptional activator for the *metE* gene [36,37], suggesting that the overexpression of *metE* is related to that of *metR*. The expression of *luxS*, encoding S-ribosyl homocysteinase, was also up-regulated in EC-2. This is notable because LuxS is involved in biofilm development in many bacteria, including *E. coli* and *S. oneidensis* MR-1 [38,39]. In addition, it was found that the *SO_1860* gene was up-regulated in EC-2 compared to ∆SO_1860, but this was due to the complete deletion of this gene in ∆SO_1860.

**Table 1:** **Differentially expressed genes in strain EC-2 (*P*** **< 0.05, |log**_**2 **_**FC| ≥ 1.0)**

**Locus tag**	**Gene**	**Putative function**	**COG description**^**1**^	**Log**_**2 **_**FC**^**2**^
**Up-regulated genes**				
*SO_0817*	*metR*	Transcriptional activator protein MetR	Transcription	3.33
*SO_0818*	*metE*	5-Methyltetrahydropteroyltriglutamate-homocysteine methyltransferase	Amino acid transport and metabolism	6.49
*SO_1101*	*luxS*	S-Ribosylhomocysteinase	Signal transduction mechanisms	1.65
*SO_1860*	*–*	Response regulator	Signal transduction mechanisms, Transcription	5.97
*SO_2404*	*aroA*	3-Phosphoshikimate 1-carboxyvinyltransferase	Amino acid transport and metabolism	1.17
*SO_3471*	*glyA*	Serine hydroxymethyltransferase	Amino acid transport and metabolism	1.55
*SO_3534*	*murJ*	Peptidoglycan lipid II flippase	General function prediction only	1.17
*SO_4189*	*–*	Hypothetical protein SO_4189	Carbohydrate transport and metabolism	1.04
*SO_4233*	*leuD*	Isopropylmalate isomerase small subunit	Amino acid transport and metabolism	1.69
*SO_4596*	*–*	Copper-transporting ATPase domain-containing protein	–	2.09
*SO_4731*	*add*	Adenosine deaminase	Nucleotide transport and metabolism	1.42
**Down-regulated genes**				
*SO_0351*	–	LuxR family DNA-binding response regulator	Signal transduction mechanisms, Transcription	-1.04
*SO_1861*	*uvrC*	Excinuclease ABC subunit C	Replication, recombination and repair	-1.30
*SO_2005*	–	DksA-type zinc finger protein	Signal transduction mechanisms	-1.16
*SO_2906*	–	Hypothetical protein SO_2906	Inorganic ion transport and metabolism	-1.92
*SO_2945*	–	Lambda phage tail fiber protein	–	-1.45
*SO_2946*	–	Lambda phage protein with carbohydrate-binding module	–	-1.10
*SO_2953*	H	Prophage LambdaSo, tail length tape meausure protein	Function unknown	-1.48
*SO_2954*	–	Hypothetical protein SO_2954	–	-1.53
*SO_2955*	–	Lambda phage minor tail protein G	–	-1.62
*SO_2956*	–	Prophage LambdaSo, major tail protein V, putative	–	-1.60
*SO_2957*	–	Lambda phage protein of unknown function	–	-1.35
*SO_2965*	–	Prophage LambdaSo, HK97 family portal protein	Function unknown	-1.24
*SO_2968*	–	Phage terminase small subunit	Replication, recombination and repair	-1.03
*SO_2969*	–	Prophage LambdaSo, holin, putative	Defense mechanisms	-1.24
*SO_4564*	–	TonB2 protein, putative	Cell wall/membrane/envelope biogenesis	-1.15

^1^Functional categorization by COG.

^2^Log_2_-transformed fold change (EC-2/∆SO_1860).

**Figure 7:**
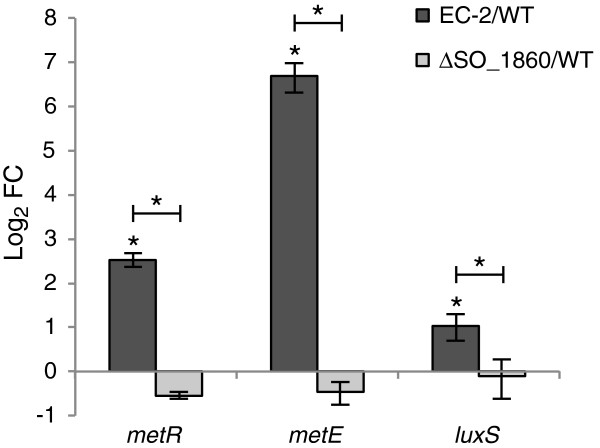
**qRT-PCR analyses of the *metR*, *metE*, and *luxS *genes in WT, EC-2, and ∆SO_1860 cells.** Results are expressed as log_2_-fold changes (FC) relative to expression levels in WT cells. The error bars represent the standard deviation calculated from at least four measurements. Asterisks indicate a statistically significant difference (*P* < 0.05) between expression levels in the three strains.

The expression levels of *metR*, *metE*, and *luxS* were also examined and compared in WT, EC-2, and ∆SO_1860 by qRT-PCR (Figure 7). It was confirmed that these three genes were overexpressed in EC-2, whereas their expression levels were not significantly different between WT and ∆SO_1860, suggesting that the overexpression of these genes is attributable to mutations specific to EC-2.

The down-regulated genes in EC-2 (Table 1) included a number of genes located within the LambdaSo prophage region (*SO_2939* to *SO_3013*) [40]. The transcriptome analysis also revealed that the *SO_1861* gene, which is located immediately downstream of *SO_1860*, was also down-regulated in EC-2. This was considered to be due to a polar effect caused by insertion of the Tn into *SO_1860*. The *SO_0351*, *SO_2005*, and *SO_4564* genes, which are categorized into the COG category “Signal transduction mechanisms” and “Cell wall/membrane/envelope biogenesis”, respectively, were also down-regulated in EC-2.

## Discussion

In this study, we isolated and characterized the novel *S. oneidensis* MR-1 mutant strain EC-2, which exhibits an increased ability to generate current in MFC and EC reactors. In addition, strain EC-2 forms flat, rough, and extraordinarily large colonies that are clearly distinct from the morphology of WT colonies (Figure 1), implying that cell surface structure and physicochemical properties are altered in this mutant. As we previously provided evidence that cell surface hydrophobicity influences the adhesiveness of *S. oneidensis* cells to graphite felt electrodes and affects current generation in MFCs [25], we also examined the adhesiveness and hydrophobicity of EC-2 cells. Compared to WT, EC-2 cells attached with higher frequency to graphite felt anodes (Figure 3) and had a more hydrophobic surface (Figure 4). Taken together, these results suggest that mutations introduced into EC-2 modified the cell surface structure and hydrophobicity, resulting in the enhanced adhesiveness of cells to graphite felt electrodes and increased current generation. A similar trend has also been observed for strain ∆SO_3177, which contains a mutation in a gene necessary for CPS synthesis and shows altered cell-surface hydrophobicity and enhanced the adhesiveness to graphite felt anodes [25]. We therefore hypothesized that genes involved in the synthesis of CPS or other cell surface structures were mutated in strain EC-2.

We determined that the *SO_1860* (*uvrY*) gene, which encodes a DNA-binding response regulator of the BarA/UvrY two-component regulatory system, was disrupted in strain EC-2 by Tn insertion. In *S. oneidensis* MR-1, *SO_1860* is involved in the transcriptional regulation of a large number of genes, including CPS biosynthesis genes [30], suggesting that disruption of *SO_1860* in strain EC-2 modified cell surface structure and led to the enhanced current generation in MFCs. The involvement of *SO_1860* in current generation was examined by constructing an in-frame deletion mutant of *SO_1860* (∆SO_1860), which was found to generate higher current in an EC, indicating the involvement of *SO_1860* in current generation. ∆SO_1860 cells also exhibited altered colony morphology and increased cell surface hydrophobicity (Figure 4), and qRT-PCR analyses demonstrated that the expression of several CPS biosynthesis genes (*SO_3172*, *SO_3177*, and *SO_3179*) was repressed (Figure 6). These results support the notion that disruption of the *SO_1860* gene affects CPS synthesis and cell surface hydrophobicity, resulting in increased current generation. However, strain EC-2 formed larger colonies, exhibited higher hydrophobicity, and generated higher current than ∆SO_1860, indicating that the disruption of *SO_1860* was not the only cause for the distinct phenotype of strain EC-2. As Southern-blotting analysis confirmed that EC-2 had a single Tn insertion in *SO_1860* (data not shown), it is likely that unknown mutations, in addition to the Tn insertion, may have been spontaneously introduced during the long-term (approximately 40 days) electrochemical cultivation of strain EC-2. Another explanation is that gene(s) located downstream of the Tn insertion site in *SO_1860* are differently expressed in EC-2. However, it is unlikely that the distinct phenotypic features of EC-2 are attributable to the decreased expression of *SO_1861* (the excinuclease ABC subunit C gene; Table 1). Further investigation, such as genome sequencing of strain EC-2, is needed to examine these hypotheses.

We examined and compared the gene expression profiles of EC-2 and ∆SO_1860 to understand the physiological differences between these two strains. qRT-PCR (Figure 6) and microarray (Table 1) analyses demonstrated that expression of CPS biosynthesis genes did not largely differ between EC-2 and ∆SO_1860, suggesting that the distinct features of EC-2 are not attributable to differential expression of CPS synthesis genes. However, we identified a number of genes that were differentially expressed between EC-2 and ∆SO_1860 (Table 1). Notably, the methionine biosynthesis genes *metR* and *metE* were highly up-regulated (10- and 90-fold, respectively) in EC-2, although it remains unclear why these genes were overexpressed. It is also interesting that the *luxS* gene was up-regulated in EC-2, as this enzyme catalyzes the conversion of S-ribosyl homocysteine to homocysteine and 4,5-dihydroxy-2,3-pentanedione (DPD), and is widely conserved in both Gram-negative and Gram-positive bacteria [41-43]. LuxS functions as an autoinducer-2 (AI-2) synthase because DPD is spontaneously converted to AI-2 [42-44]. LuxS is also involved in the activated methyl cycle (AMC) [38,43] which is responsible for the synthesis of homocysteine, methionine, and S-adenosylmethionine (SAM), a major methyl donor source that is utilized for various cellular processes including the methylation of DNA and methyl-accepting chemotaxis proteins [43,45]. MetE (methionine synthase) and MetR (transcriptional activator for *met* genes) are also involved in the AMC. In *S. oneidensis*, disruption of *luxS* negatively affects biofilm development on solid surfaces by interrupting the AMC [39]. It has been also reported that a *luxS*-complemented (overexpressing) mutant of *E. coli* was deficient in pili production and formed a thicker biofilm than the WT strain, phenotypes that were suggested to be due to the depletion of SAM resulting from elevated *luxS* expression [38]. In addition, a *metR* mutant (∆*metR*) of *Pseudomonas aeruginosa* exhibited altered colony morphology due to a severe defect in swarming motility [46], suggesting that the AMC is associated with cell motility and colony morphology. It is therefore possible that the overexpression of AMC-associated genes in EC-2 influences cell surface structure or motility, resulting in the altered colony morphology and increased adhesiveness of cells to electrodes. Studies are underway to investigate this possibility.

The function and expression of the *metR*, *metE*, and *luxS* genes are considered to be closely related, because homocysteine, one of the products of LuxS, acts as a co-regulator for MetR and stimulates the transcription of *metE*[47]. We found a potential MetR-binding site in the upstream intergenic region of the MR-1 *luxS* gene (5’-TGAGATGATTTCA-3’) that closely matches the consensus MetR-binding sequence reported in *E. coli* and other bacteria (5’-TGAANNANNTTCA-3’) [48]. A similar sequence was also identified in the intergenic region between *metE* and *metR* (5’-TGAGCGAAATTCA-3’). These findings suggest the possibility that MetR regulates the expression of *luxS*, as well as that of *met* genes, in *S. oneidensis* MR-1. A putative MetR-binding sequence was also found upstream of *glyA* (5’-TGAGGTGCATTCA-3’). Because MetR activates the transcription of *glyA* in *E. coli*[49], it is likely that the overexpression of *metR* in EC-2 resulted in the increased expression of *glyA*. In addition to these AMC-related genes, the microarray analysis detected 20 genes other than *SO_1860* and *SO_1861* that were differentially regulated in EC-2, including 10 genes located within the LambdaSo prophage region (Table 1). Although it has been reported that prophage-mediated cell lysis enhances biofilm formation in *S. oneidensis* MR-1 [40], the regulation and involvement of these genes in the observed phenotype of EC-2 is unknown.

## Conclusions

The present study indicates that *SO_1860* (*uvrY*) and other biofilm formation-related genes, including those involved in CPS biosynthesis and the AMC, play important roles in determining colony morphology, cell surface properties, and current-generating capability of *S. oneidensis* MR-1. Thus, it might be possible to control the adhesion of cells to electrodes by altering the expression of these genes, and thereby increase the efficiency of BESs. Although the present study focused on the altered gene expression profiles of strain EC-2, proteomic and metabolomic approaches will also provide useful information for understanding the distinct features of this mutant. Future studies will be conducted to elucidate the mechanisms underlying the increased current-generating capability of strain EC-2.

## Methods

### Bacterial strains and plasmids

*S. oneidensis* MR-1 was obtained from American Type Culture Collection (ATCC). *E. coli* strains [8] were routinely cultured in Luria-Bertani (LB) medium at 37°C. The *E. coli* mating strain (WM6026) required supplementation of the medium with 100 μg ml^-1^ 2,6-diaminopimelic acid (DAP) for growth. *Shewanella* strains were cultured at 30°C in either LB or lactate minimal medium [25] supplemented with 0.2 g liter^-1^ casamino acids and 10 ml liter^-1^ each of amino acid and trace mineral solutions (LMM). When necessary, 50 μg ml^-1^ kanamycin (Km) was added to culture media. Agar plates contained 1.6% Bacto agar (Difco).

### Construction of a mutant library

Random Tn mutagenesis of *S. oneidensis* MR-1 was performed by filter mating with *E. coli* WM6026 harboring the suicide plasmid pBSL180 [50], which contained mini-Tn10Km^r^, according to a previously described method [25,26]. After transformed cells were grown on LB-agar plates containing DAP at 30°C for 8 h, the cells were washed in 10 mM MgSO_4_ and then aerobically grown in LMM containing Km for 24 h.

### Selection of mutants

Selection of mutants from the random Tn-insertion library was carried out using a previously described method [26] with slight modifications. Briefly, a cylindrical electrochemical cell (EC; 500 ml capacity) equipped with a graphite-felt working electrode (WE; 50 cm^2^; GF-80-3 F, Sohgoh Carbon), air diffusion-type counter electrode (approximately 20 cm^2^; 0.7 mg platinum/cm^2^; and four polytetrafluoroethylene layers) was constructed as described elsewhere [51], and Ag/AgCl was used as the reference electrode (HX-R5, Hokuto Denko). The EC was filled with 450 ml LMM, purged with pure nitrogen gas, and then inoculated with the mutant library at a final cell concentration of approximately 2 × 10^6^ ml^-1^. The EC was incubated at 30°C for 40 days under constant agitation, and the working electrode was poised at +0.2 V vs. the Ag/AgCl reference electrode using a potentiostat (Multipotentiostat 2092, Toho Giken). Current was monitored using the potentiostat, and current density (A cm^-2^) was calculated based on the anode projection area (50 cm^2^). When the current density fell below 0.1 mA, lactate was injected into the EC at a final concentration of 10 mM.

### Isolation of mutants

After the 40-day electrochemical selection in the EC, the electrolyte was collected, serially diluted, and spread on agar plates containing LB supplemented with Km. Colonies that formed on the agar plates were randomly selected and purified by re-streaking for isolated colonies. Isolated mutants were grown in LB medium supplemented with Km and stored at -80°C in 15% (v/v) glycerol. Tn-insertion sites of the isolated mutants were identified according to a method described previously [26].

### Gene disruption

In-frame disruption of the *SO_1860* gene in strain MR-1 was performed using a two-step homologous recombination method with suicide plasmid pSMV-10, as described previously [8,25,52]. Briefly, a 1.6-kb fusion product, consisting of an upstream (768 bp) and downstream (798 bp) sequence of the *SO_1860* gene (768 bp) joined by an 18-bp linker sequence, was constructed by PCR and *in-vitro* extension using the primers listed in Additional file 5: Table S1. The amplified fusion product was ligated into the SpeI site of pSMV10, generating pSMV-1860, which was then introduced into MR-1 by filter mating with *E. coli* WM6026. Transconjugants (single-crossover clones) were selected on LB plates containing Km and further cultivated for 20 h in LB medium lacking antibiotics. The cultures were then spread onto LB plates containing 10% (w/v) sucrose to isolate Km-sensitive double-crossover mutants. Disruption of the *SO_1860* gene in the obtained strains was confirmed by PCR. One representative mutant strain in which the *SO_1860* gene was disrupted in-frame was selected and designated ∆SO_1860.

### Evaluation of mutants in MFCs and ECs

In MFC experiments, microbial current generation was measured using a single-chamber MFC equipped with a graphite felt anode (50 cm^2^; GF-80-3 F) and air cathode (approximately 20 cm^2^; 0.7 mg platinum/cm^2^; and four polytetrafluoroethylene layers), as previously described [25]. Bacterial cells were inoculated into the MFC chamber, which contained 450 ml LMM supplemented with 10 mM lactate, at an initial optical density at 600 nm (OD_600_) of 0.005. Upon depletion of lactate, a 4.5 M stock solution of lactate was injected into the reactor to increase the concentration of lactate to 10 mM. The anode and cathode were connected via electric wires and an external resistor (100 Ω), and the voltage across the resistor was measured using a voltage data logger (HA-1510, Graphtec). Current (*I* [A]) was calculated using the equation: *I* = *E*/*R*, where E [V] is the cell voltage and R [Ω] is the resistance. Current density (A cm^-2^) was calculated using the anode projection area (50 cm^2^). A polarization curve was generated using a potentiostat (HSV-100; Hokuto Denko), from which the maximum power density (*P*_max_ [W cm^-2^]) and short-circuit current (*I*_sc_ [A cm^-2^]) were obtained as described elsewhere [53]. Reproducibility was examined in at least three independent measurements, and typical data are shown here. The protein contents in planktonic cells, anode biofilms, and cathode biofilms in MFC reactors were determined using a BCA protein assay Kit (Pierce) according to a method described previously [25].

A low-volume (18 ml capacity), single-chamber EC equipped with a graphite felt working electrode (2.3 cm^2^; poised at +0.2 V *vs.* an Ag/AgCl reference electrode [HX-R5, Hokuto Denko]) was used to monitor and compare current generation by WT, EC-2, and ∆SO_1860 cells. A platinum wire (5 cm, φ0.3 mm; Nilaco) was used as the counter electrode. Bacterial cells were inoculated into the EC chamber, which containing 15 ml LMM supplemented with 10 mM lactate, at an initial OD_600_ of 0.01. Current was monitored using a potentiostat (HA-1510; Hokuto Denko), and current density (A cm^-2^) was calculated based on the anode projection area (2.3 cm^2^). Reproducibility was examined in at least three independent measurements.

### Hydrophobicity assay

Cell hydrophobicity was analyzed by the bacterial adhesion to hydrocarbon method (BATH), as described previously [25,54]. Briefly, *Shewanella* cells were suspended in 2.4 ml of 0.15 M NaCl at an OD_600_ of 0.3 (approximately 3 × 10^8^ CFU ml^-1^) and vortexed for 60 s in the presence of 0.4 ml hexadecane. The mixture was allowed to stand for 15 min at room temperature to ensure that the two phases were completely separated before a 1-ml sample was removed from the aqueous phase for measuring the OD_600_. The percentage of cells transferred to the hexadecane phase was subsequently calculated using the equation: affinity (%) = 100 × [1 - (*A*/*A*_0_)], where *A*_0_ is the OD_600_ of the bacterial suspension before mixing with hexadecane and A is the OD_600_ after mixing. All measurements were performed in at least six replicates, and data were statistically analyzed by one-way analysis of variance (ANOVA) in combination with Holm’s multiple-comparison test using js-STAR software (http://www.kisnet.or.jp/nappa/software/star/). A *P*-value of 0.05 was considered statistically significant.

### RNA extraction

*Shewanella* cells were grown anaerobically in LMM (containing 15 mM lactate) supplemented with 20 mM fumarate as the electron acceptor, and cells were harvested at the early stationary growth phase (OD_600_ of 0.16-0.18). Total RNA was extracted using Trizol reagent (Invitrogen) following the manufacturer’s instructions and subsequently purified using an RNeasy Mini Kit and RNase-Free DNase Set (Qiagen). The quality of extracted RNA was evaluated using an Agilent 2100 Bioanalyzer with RNA 6000 Pico reagents and RNA Pico Chips (Agilent Technologies) according to the manufacturer’s instructions. The purified RNA was then used for qRT-PCR and microarray transcriptome analysis.

### qRT-PCR

qRT-PCR was performed according to a method described previously [55,56] with slight modifications. RT and subsequent quantitative PCR were conducted using a LightCycler 1.5 instrument (Roche) following the manufacturer’s instructions. The PCR mixture (20 μl) contained 1 μl diluted RNA (150 ng for *luxS*, and 15 ng for other genes), 1.3 μl of 50 mM Mn (OAc)_2_ solution, 7.5 μl LightCycler RNA Master SYBR Green I (Roche), and 0.15 μM of the primers listed in Supplemental Table 1. A standard curve was drawn using serial dilutions of PCR fragments of each gene. Specificity of the quantitative PCR was verified by dissociation-curve analysis. The expression levels of the target genes were normalized based on the expression level of the 16S rRNA gene. All measurements were performed in quadruplicate at a minimum, and data were statistically analyzed by one-way ANOVA with Bonferroni’s multiple-comparison test using SAS software (version 9.3, SAS Institute). A *P*-value of 0.05 was considered statistically significant.

### Microarray analysis

A custom 60-mer oligonucleotide microarray (8 × 15 K; Agilent Technologies) containing (maximally) three different probes per gene was designed using the annotated chromosomal and megaplasimd sequences of *S. oneidensis* MR-1 (Genbank accession nos. AE014299 and AE014300, respectively). Specific oligonucleotide probes were designed for 4,772 genes (corresponding to 96.6% of the total annotated genes) using the eArray protocol (Agilent Technologies) and fabricated on slide glass by SurePrint technology (Agilent Technologies). Fluorescence labeling of cDNA, and hybridization and scanning of hybridized arrays were performed according to the manufacturer’s protocols for gene expression arrays for prokaryotes (Agilent One-Color Microarray-Based Prokaryote Analysis, version 1.4, http://www.chem.agilent.com). Briefly, cyanine 3 (Cy3)-labeled cDNA was synthesized from 5 μg total RNA using the FairPlay III Microarray Labeling KitStratagene with CyDye Cy3 mono-Reactive Dye (GE Healthcare). The labeled cDNA was quantified using a NanoDrop ND-1000 Spectrophotometer (Thermo Scientific). For each array, 20 μl of the purified Cy3-labeled cDNA (20 ng/μl) was mixed with 5 μl of 10× Blocking Agent and 25 μl of 2× GEx Hybridization Buffer HI-RPM. The resultant mixture (40 μl) was hybridized to the array at 65°C for 17 h. After hybridization, each slide was washed with Gene Expression Wash Buffer I (Agilent Technologies) at room temperature for 1 min, followed by Gene Expression Wash Buffer II (Agilent Technologies) at 37°C for 1 min. Slides were air dried and then scanned using an Agilent DNA Microarray Scanner at 5-μm resolution. Data acquisition was performed using the Feature Extraction Software version 8.1 (Agilent Technologies). Gene expression data (n = 3 biological replicates) were normalized and statistically analyzed using GeneSpring GX version 11.5 (Agilent Technologies). The unpaired Student’s t-test and the Benjamini-Hochberg false discovery rate correction were used for statistical analysis. Differential expression for each probe was considered statistically significant when the fold change (FC) was ≥ 2.0 or ≤ 0.5 (|log_2_ FC| ≥ 1.0) at a *P*-value of < 0.05. The average FC was calculated for each gene from the values of the probes with altered expression. The microarray data have been deposited in the NCBI Gene Expression Omnibus (GEO) under the accession number GSE50443.

## Abbreviations

EET: Extracellular electron transfer; CPS: Cell-surface polysaccharide; EC: Electrochemical cell; BES: Bioelectrochemical system; MFC: Microbial fuel cell; OM-cyt: Outer-membrane cytochrome; Tn: Transposon; LB: Luria-Bertani; DAP: 2:6-Diaminopimelic acid; LMM: Lactate minimal medium; WT: Wild-type; DPD: 4,5-Dihydroxy-2,3-pentanedione; FC: Fold change; AMC: Activated methyl cycle; SAM: S-adenosylmethionine.

## Competing interests

The authors declare that they have no competing interests.

## Authors’ contributions

AK participated in the design of the study, performed the molecular genetic studies, and drafted the manuscript. HO participated in the evaluation of mutants, qRT-PCR, and microarray analysis. NT participated in the isolation of mutants. KH participated in the design and coordination of the study. KW conceived of the study, participated in its design and coordination, and performed manuscript editing. All authors read and approved the final manuscript.

## Supplementary Material

Additional file 1: Figure S1 Current generation in an EC inoculated with a random transposon mutant library of *S. oneidensis* MR-1. Click here for file

Additional file 2: Figure S2 Colonies of mutants with altered morphology isolated after electrochemical enrichment. Mutants with distinct colony morphology were picked and further cultivated on a LB agar plate for 2 days. An arrowhead indicates a colony similar in size to that of WT. Click here for file

Additional file 3: Figure S3 Current generation by strain EC-2 (grey line) and WT (black line) in single-chamber MFCs. An arrowhead indicates the time point at which polarization (Figure 2A) and power (Figure 2B) curves were measured. Reproducibility was examined in at least three independent operations, and typical data are shown. Click here for file

Additional file 4: Figure S4 Comparison of transcriptional changes in strains EC-2 and ∆SO_1860 as determined by microarray and qRT-PCR analyses. Log_2_-transformed fold changes (Log_2_ FC) in the expression levels (EC-2/∆SO_1860) of 5 selected genes determined by microarray analysis were plotted against the values determined by qRT-PCR. Click here for file

Additional file 5: Table S1 Primers used in this study. Click here for file

## References

[B1] Fredrickson JK Romine MF Beliaev AS Auchtung JM Driscoll ME Gardner TS Nealson KH Osterman AL Pinchuk G Reed JL Rodionov DA Rodrigues JLM Saffarini DA Serres MH Spormann AM Zhulin IB Tiedje JM Towards environmental systems biology of *Shewanella* Nat Rev Microbiol 2008 6 592 603 18604222 10.1038/nrmicro1947

[B2] Nealson KH Saffarini D Iron and manganese in anaerobic respiration: environmental significance, physiology, and regulation Annu Rev Microbiol 1994 48 311 343 7826009 10.1146/annurev.mi.48.100194.001523

[B3] Liu C Gorby YA Zachara JM Fredrickson JK Brown CF Reduction kinetics of Fe (III), Co (III), U (VI), Cr (VI), and Tc (VII) in cultures of dissimilatory metal-reducing bacteria Biotechnol Bioeng 2002 80 637 649 12378605 10.1002/bit.10430

[B4] Hau HH Gilbert A Coursolle D Gralnick JA Mechanism and Consequences of anaerobic respiration of cobalt by *Shewanella oneidensis* strain MR-1 Appl Environ Microbiol 2008 74 6880 6886 18836009 10.1128/AEM.00840-08 PMC2583509

[B5] Carpentier W Sandra K De Smet I Brigé A De Smet L Van Beeumen J Microbial reduction and precipitation of vanadium by *Shewanella oneidensis* Appl Environ Microbiol 2003 69 3636 3639 12788772 10.1128/AEM.69.6.3636-3639.2003 PMC161487

[B6] Hau HH Gralnick JA Ecology and biotechnology of the genus *Shewanella* Annu Rev Microbiol 2007 61 237 258 18035608 10.1146/annurev.micro.61.080706.093257

[B7] Kim BH Kim HJ Hyun MS Park DH Direct electrode reaction of Fe (III)-reducing bacterium, *Shewanella putrefaciens* J Microbiol Biotechnol 1999 9 127 131

[B8] Newton GJ Mori S Nakamura R Hashimoto K Watanabe K Analyses of current-generating mechanisms of *Shewanella loihica* PV-4 and *Shewanella oneidensis* MR-1 in microbial fuel cells Appl Environ Microbiol 2009 75 7674 7681 19837834 10.1128/AEM.01142-09 PMC2794086

[B9] Ross DE Flynn JM Baron DB Gralnick JA Bond DR Towards electrosynthesis in *Shewanella*: energetics of reversing the Mtr pathway for reductive metabolism PLoS One 2011 6 e16649 21311751 10.1371/journal.pone.0016649 PMC3032769

[B10] Flynn JM Ross DE Hunt KA Bond DR Gralnick JA Enabling unbalanced fermentations by using engineered electrode-interfaced bacteria MBio 2010 1 1 8 10.1128/mBio.00190-10 PMC2975363 21060736

[B11] Myers CR Nealson KH Bacterial manganese reduction and growth with manganese oxide as the sole electron acceptor Science 1988 240 1319 1321 17815852 10.1126/science.240.4857.1319

[B12] Heidelberg JF Paulsen IT Nelson KE Gaidos EJ Nelson WC Read TD Eisen JA Seshadri R Ward N Methe B Clayton RA Meyer T Tsapin A Scott J Beanan M Brinkac L Daugherty S DeBoy RT Dodson RJ Durkin a S Haft DH Kolonay JF Madupu R Peterson JD Umayam LA White O Wolf AM Vamathevan J Weidman J Impraim M Genome sequence of the dissimilatory metal ion-reducing bacterium *Shewanella oneidensis* Nat Biotechnol 2002 20 1118 1123 12368813 10.1038/nbt749

[B13] Daraselia N Dernovoy D Tian Y Borodovsky M Tatusov R Tatusova T Reannotation of *Shewanella oneidensis* genome OMICS 2003 7 171 175 14506846 10.1089/153623103322246566

[B14] Bretschger O Obraztsova A Sturm CA Chang IS Gorby YA Reed SB Culley DE Reardon CL Barua S Romine MF Zhou J Beliaev AS Bouhenni R Saffarini D Mansfeld F Kim BH Fredrickson JK Nealson KH **Current production and metal oxide reduction by **** *Shewanella oneidensis * ****MR-1 wild type and mutants.** Appl Environ Microbiol 2007 73 7003 7012 17644630 10.1128/AEM.01087-07 PMC2074945

[B15] Shi L Squier TC Zachara JM Fredrickson JK Respiration of metal (hydr)oxides by *Shewanella* and *Geobacter*: a key role for multihaem c-type cytochromes Mol Microbiol 2007 65 12 20 17581116 10.1111/j.1365-2958.2007.05783.x PMC1974784

[B16] Gorby YA Yanina S McLean JS Rosso KM Moyles D Dohnalkova A Beveridge TJ Chang IS Kim BH Kim KS Culley DE Reed SB Romine MF Saffarini DA Hill EA Shi L Elias DA Kennedy DW Pinchuk G Watanabe K Ishii S Logan B Nealson KH Fredrickson JK Electrically conductive bacterial nanowires produced by *Shewanella oneidensis* strain MR-1 and other microorganisms Proc Natl Acad Sci U S A 2006 103 11358 11363 16849424 10.1073/pnas.0604517103 PMC1544091

[B17] El-Naggar MY Wanger G Leung KM Yuzvinsky TD Southam G Yang J Lau WM Nealson KH Gorby YA Electrical transport along bacterial nanowires from *Shewanella oneidensis* MR-1 Proc Natl Acad Sci U S A 2010 107 18127 18131 20937892 10.1073/pnas.1004880107 PMC2964190

[B18] Marsili E Baron DB Shikhare ID Coursolle D Gralnick JA Bond DR *Shewanella secretes* flavins that mediate extracellular electron transfer Proc Natl Acad Sci U S A 2008 105 3968 3973 18316736 10.1073/pnas.0710525105 PMC2268775

[B19] von Canstein H Ogawa J Shimizu S Lloyd JR Secretion of flavins by *Shewanella* species and their role in extracellular electron transfer Appl Environ Microbiol 2008 74 615 623 18065612 10.1128/AEM.01387-07 PMC2227709

[B20] Watanabe K Manefield M Lee M Kouzuma A Electron shuttles in biotechnology Curr Opin Biotechnol 2009 20 633 641 19833503 10.1016/j.copbio.2009.09.006

[B21] Okamoto A Hashimoto K Nealson KH Nakamura R Rate enhancement of bacterial extracellular electron transport involves bound flavin semiquinones Proc Natl Acad Sci U S A 2013 110 7856 7861 23576738 10.1073/pnas.1220823110 PMC3651484

[B22] Saffarini DA Schultz R Beliaev A Involvement of cyclic AMP (cAMP) and cAMP receptor protein in anaerobic respiration of *Shewanella oneidensis* J Bacteriol 2003 185 3668 3671 12775705 10.1128/JB.185.12.3668-3671.2003 PMC156221

[B23] Charania MA Brockman KL Zhang Y Banerjee A Pinchuk GE Fredrickson JK Beliaev AS Saffarini DA Involvement of a membrane-bound class III adenylate cyclase in regulation of anaerobic respiration in *Shewanella oneidensis* MR-1 J Bacteriol 2009 191 4298 4306 19395492 10.1128/JB.01829-08 PMC2698484

[B24] Covington ED Gelbmann CB Kotloski NJ Gralnick JA An essential role for UshA in processing of extracellular flavin electron shuttles by *Shewanella oneidensis* Mol Microbiol 2010 78 519 532 20807196 10.1111/j.1365-2958.2010.07353.x

[B25] Kouzuma A Meng XY Kimura N Hashimoto K Watanabe K Disruption of the putative cell surface polysaccharide biosynthesis gene SO3177 in *Shewanella oneidensis* MR-1 enhances adhesion to electrodes and current generation in microbial fuel cells Appl Environ Microbiol 2010 76 4151 4157 20453127 10.1128/AEM.00117-10 PMC2897461

[B26] Tajima N Kouzuma A Hashimoto K Watanabe K Selection of *Shewanella oneidensis* MR-1 gene-knockout mutants that adapt to an electrode-respiring condition Biosci Biotechnol Biochem 2011 75 2229 2233 22056453 10.1271/bbb.110539

[B27] Pernestig AK Melefors O Georgellis D Identification of UvrY as the cognate response regulator for the BarA sensor kinase in *Escherichia coli* J Biol Chem 2001 276 225 231 11022030 10.1074/jbc.M001550200

[B28] Wei B Shin S LaPorte D Wolfe AJ Romeo T Global regulatory mutations in *csrA* and *rpoS* cause severe central carbon stress in *Escherichia coli* in the presence of acetate J Bacteriol 2000 182 1632 1640 10692369 10.1128/jb.182.6.1632-1640.2000 PMC94461

[B29] Pernestig AK Georgellis D Romeo T Suzuki K Tomenius H Normark S Melefors O The *Escherichia coli* BarA-UvrY two-component system is needed for efficient switching between glycolytic and gluconeogenic carbon sources J Bacteriol 2003 185 843 853 12533459 10.1128/JB.185.3.843-853.2003 PMC142795

[B30] Binnenkade L Lassak J Thormann KM Analysis of the BarA/UvrY Two-Component System in *Shewanella oneidensis* MR-1 PLoS One 2011 6 e23440 21931597 10.1371/journal.pone.0023440 PMC3171408

[B31] Müller J Shukla S Jost KA Spormann AM The *mxd* operon in *Shewanella oneidensis* MR-1 is induced in response to starvation and regulated by ArcS/ArcA and BarA/UvrY BMC Microbiol 2013 13 119 23705927 10.1186/1471-2180-13-119 PMC3691769

[B32] Boyd A Chakrabarty AM *Pseudomonas aeruginosa* biofilms: role of the alginate exopolysaccharide J Ind Microbiol 1995 15 162 168 8519473 10.1007/BF01569821

[B33] Davey ME Duncan MJ Enhanced biofilm formation and loss of capsule synthesis: deletion of a putative glycosyltransferase in *Porphyromonas gingivalis* J Bacteriol 2006 188 5510 5523 16855241 10.1128/JB.01685-05 PMC1540017

[B34] Korenevsky A Beveridge TJ The surface physicochemistry and adhesiveness of *Shewanella* are affected by their surface polysaccharides Microbiology 2007 153 1872 1883 17526844 10.1099/mic.0.2006/003814-0

[B35] Tatusov RL Fedorova ND Jackson JD Jacobs AR Kiryutin B Koonin EV Krylov DM Mazumder R Mekhedov SL Nikolskaya AN Rao BS Smirnov S Sverdlov AV Vasudevan S Wolf YI Yin JJ Natale DA The COG database: an updated version includes eukaryotes BMC Bioinformatics 2003 4 41 12969510 10.1186/1471-2105-4-41 PMC222959

[B36] Fritsch PS Urbanowski ML Stauffer GV **Role of the RNA polymerase α subunits in MetR-dependent activation of **** *metE * ****and **** *metH* ****: important residues in the C-terminal domain and orientation requirements within RNA polymerase.** J Bacteriol 2000 182 5539 5550 10986259 10.1128/jb.182.19.5539-5550.2000 PMC110999

[B37] Weissbach H Brot N Regulation of methionine synthesis in *Escherichia coli* Mol Microbiol 1991 5 1593 1597 1943695 10.1111/j.1365-2958.1991.tb01905.x

[B38] Niu C Robbins CM Pittman KJ Osborn JL Stubblefield BA Simmons RB Gilbert ES LuxS influences *Escherichia coli* biofilm formation through autoinducer-2-dependent and autoinducer-2-independent modalities FEMS Microbiol Ecol 2013 83 778 791 23078586 10.1111/1574-6941.12034

[B39] Learman DR Yi H Brown SD Martin SL Geesey GG Stevens AM Hochella MF Involvement of *Shewanella oneidensis* MR-1 LuxS in biofilm development and sulfur metabolism Appl Environ Microbiol 2009 75 1301 1307 19124589 10.1128/AEM.01393-08 PMC2648156

[B40] Gödeke J Paul K Lassak J Thormann KM Phage-induced lysis enhances biofilm formation in *Shewanella oneidensis* MR-1 ISME J 2011 5 613 626 20962878 10.1038/ismej.2010.153 PMC3105746

[B41] Winzer K Hardie KR Burgess N Doherty N Kirke D Holden MTG Linforth R Cornell KA Taylor AJ Hill PJ Williams P LuxS: its role in central metabolism and the in vitro synthesis of 4-hydroxy-5-methyl-3(2H)-furanone Microbiology 2002 148 909 922 11932438 10.1099/00221287-148-4-909

[B42] Pei D Zhu J Mechanism of action of S-ribosylhomocysteinase (LuxS) Curr Opin Chem Biol 2004 8 492 497 15450491 10.1016/j.cbpa.2004.08.003

[B43] Vendeville A Winzer K Heurlier K Tang CM Hardie KR Making “sense” of metabolism: autoinducer-2, LuxS and pathogenic bacteria Nat Rev Microbiol 2005 3 383 396 15864263 10.1038/nrmicro1146

[B44] Xavier KB Bassler BL LuxS quorum sensing: more than just a numbers game Curr Opin Microbiol 2003 6 191 197 12732311 10.1016/s1369-5274(03)00028-6

[B45] Chiang PK Gordon RK Tal J Zeng GC Doctor BP Pardhasaradhi K McCann PP S-Adenosylmethionine and methylation FASEB J 1996 10 471 480 8647346

[B46] Yeung ATY Torfs ECW Jamshidi F Bains M Wiegand I Hancock REW Overhage J Swarming of *Pseudomonas aeruginosa* is controlled by a broad spectrum of transcriptional regulators, including MetR J Bacteriol 2009 191 5592 5602 19592586 10.1128/JB.00157-09 PMC2737960

[B47] Urbanowski ML Stauffer GV Role of homocysteine in *metR*-mediated activation of the *metE* and *metH* genes in *Salmonella typhimurium* and *Escherichia coli* J Bacteriol 1989 171 3277 3281 2656646 10.1128/jb.171.6.3277-3281.1989 PMC210046

[B48] Chatterjee J Miyamoto CM Zouzoulas A Lang BF Skouris N Meighen EA MetR and CRP bind to the *Vibrio harveyi lux* promoters and regulate luminescence Mol Microbiol 2002 46 101 111 12366834 10.1046/j.1365-2958.2002.03128.x

[B49] Plamann MD Stauffer GV **Regulation of the **** *Escherichia coli glyA * ****gene by the **** *metR * ****gene product and homocysteine.** J Bacteriol 1989 171 4958 4962 2670901 10.1128/jb.171.9.4958-4962.1989 PMC210303

[B50] Alexeyev MF Shokolenko IN Mini-Tn10 transposon derivatives for insertion mutagenesis and gene delivery into the chromosome of gram-negative bacteria Gene 1995 160 59 62 7628717 10.1016/0378-1119(95)00141-r

[B51] Cheng S Liu H Logan BE Increased power generation in a continuous flow MFC with advective flow through the porous anode and reduced electrode spacing Environ Sci Technol 2006 40 2426 2432 16646485 10.1021/es051652w

[B52] Saltikov CW Newman DK Genetic identification of a respiratory arsenate reductase Proc Natl Acad Sci U S A 2003 100 10983 10988 12939408 10.1073/pnas.1834303100 PMC196913

[B53] Watanabe K Recent developments in microbial fuel cell technologies for sustainable bioenergy J Biosci Bioeng 2008 106 528 536 19134546 10.1263/jbb.106.528

[B54] Rosenberg M Gutnick D Rosenberg E Adherence of bacteria to hydrocarbons: a simple method for measuring cell-surface hydrophobicity FEMS Microbiol Lett 1980 9 29 33

[B55] Kouzuma A Hashimoto K Watanabe K **Influences of aerobic respiration on current generation by **** *Shewanella oneidensis * ****MR-1 in single-chamber microbial fuel cells.** Biosci Biotechnol Biochem 2012 76 270 275 22313754 10.1271/bbb.110633

[B56] Kouzuma A Hashimoto K Watanabe K Roles of siderophore in manganese-oxide reduction by *Shewanella oneidensis* MR-1 FEMS Microbiol Lett 2012 326 91 98 22092340 10.1111/j.1574-6968.2011.02444.x

