# Supplementary figures and images for: Electrochemical selection and characterization of a high current-generating Shewanella oneidensis mutant with altered cell-surface morphology and biofilm-related gene expression
Source: BMC Microbiol. 2014 Jul 16;14:190. doi: 10.1186/1471-2180-14-190 (PMC4112983; doi:10.1186/1471-2180-14-190)

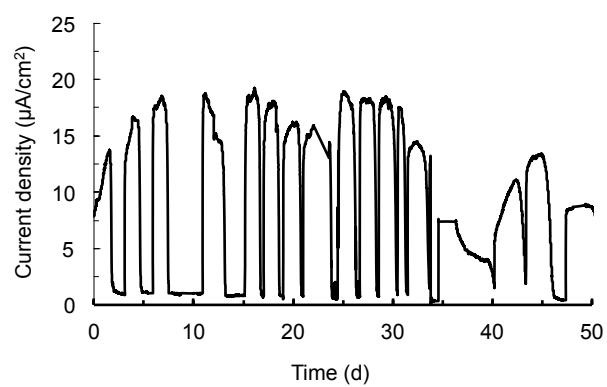

Supplement: Additional file 1: Figure S1 — Current generation in an EC inoculated with a random transposon mutant library of S. oneidensis MR-1. [file 1471-2180-14-190-S1.pdf]

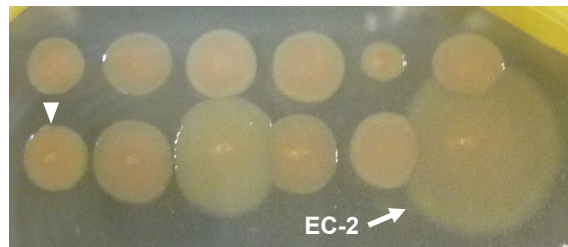

Supplement: Additional file 2: Figure S2 — Colonies of mutants with altered morphology isolated after electrochemical enrichment. Mutants with distinct colony morphology were picked and further cultivated on a LB agar plate for 2 days. An arrowhead indicates a colony similar in size to that of WT. [file 1471-2180-14-190-S2.pdf]

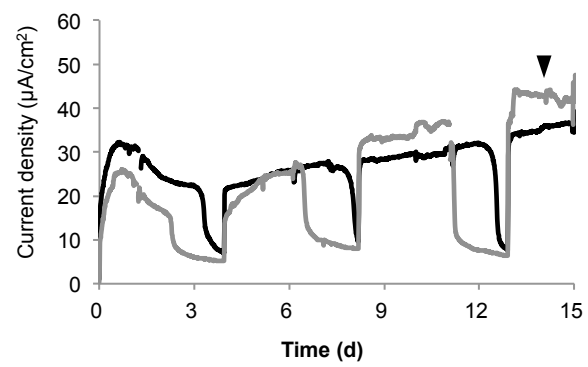

Supplement: Additional file 3: Figure S3 — Current generation by strain EC-2 (grey line) and WT (black line) in single-chamber MFCs. An arrowhead indicates the time point at which polarization (Figure 2A) and power (Figure 2B) curves were measured. Reproducibility was examined in at least three independent operations, and typical data are shown. [file 1471-2180-14-190-S3.pdf]

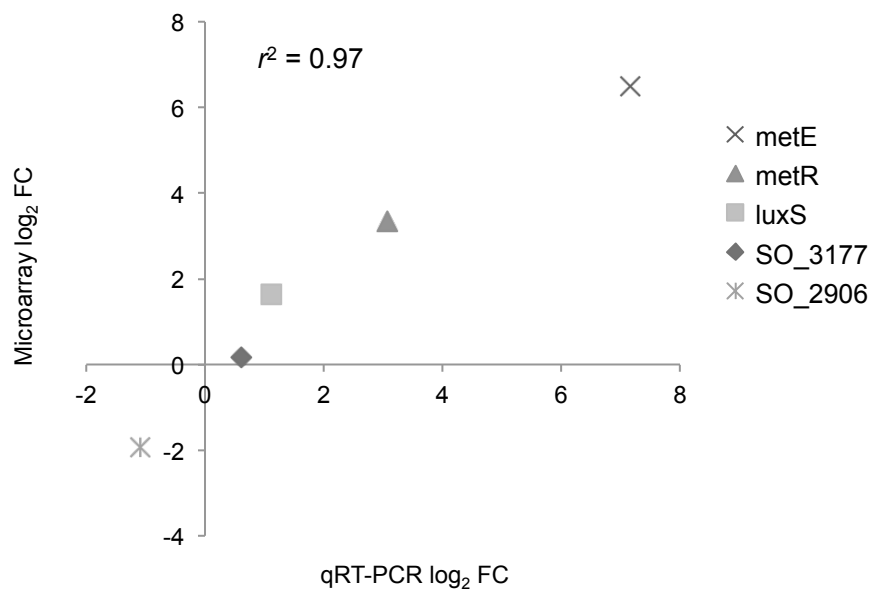

Supplement: Additional file 4: Figure S4 — Comparison of transcriptional changes in strains EC-2 and ∆SO_1860 as determined by microarray and qRT-PCR analyses. Log2-transformed fold changes (Log2 FC) in the expression levels (EC-2/∆SO_1860) of 5 selected genes determined by microarray analysis were plotted against the values determined by qRT-PCR. [file 1471-2180-14-190-S4.pdf]
