# Supplementary material for: Electrochemical selection and characterization of a high current-generating Shewanella oneidensis mutant with altered cell-surface morphology and biofilm-related gene expression
Source: BMC Microbiol. 2014 Jul 16;14:190. doi: 10.1186/1471-2180-14-190 (PMC4112983; doi:10.1186/1471-2180-14-190)
Supplement: Additional file 5: Table S1 — Primers used in this study. [file 1471-2180-14-190-S5.pdf]

Table S1. Primers used in this study.

Primer	Sequence (5' to 3')	Modification, for use
1860_F-O	TTAGCGAGCTAGAATAGGGC	SO_1860 disruption
1860_5-O_SpeI	ATGACTAGTACAGTCTTGGGCGAGATGTA	SpeI, SO_1860 disruption
1860_5-I	GGTGTTAGCCACTGAAGCTACCAGCTCATGATCGTC	Linker sequence, SO_1860 disruption
1860_3-I	GCTTCAGTGGCTAACACCACCCGTTTAGCGATCCGC	Linker sequence, SO_1860 disruption
1860_3-O-SpeI	ACGACTAGTTCTTCCATCTTCGCCACCAG	SpeI, SO_1860 disruption
1860_R-O	TGCTCATAGGCTTGCTGCTC	SO_1860 disruption
qRT-16S-F	AGCGCAACCCCTATCCTTAT	qRT-PCR for 16S rRNA gene
qRT-16S-R	CGTAAGGGCCATGATGACTT	qRT-PCR for 16S rRNA gene
qRT-SO_3172-F	GCTCATCGTCACCATCATTG	qRT-PCR for SO_3172
qRT-SO_3172-R	CCGATGCGGTATCCTTACTC	qRT-PCR for SO_3172
qRT-SO3177-F	TGTAAACGGAACGAGGATCA	qRT-PCR for SO_3177
qRT-SO3177-R	TTAGCAAGTGCCCAATAGCC	qRT-PCR for SO_3177
qRT-luxS-F	CATATGACGACCCCAAAAGG	qRT-PCR for *luxS* (SO_1101)
qRT-luxS-R	ATAAAGCCCGCAAACAAATG	qRT-PCR for *luxS* (SO_1101)
qRT-metE-F	GAAGGTGTGGGCTTTACCAA	qRT-PCR for *metE* (SO_0818)
qRT-metE-R	AATCAACCGTCATGGCTTTC	qRT-PCR for *metE* (SO_0818)
qRT-metR-F	TCAGCTAAGGGTTGGGATTG	qRT-PCR for *metR* (SO_0817)
qRT-metR-R	TTCTAGGGCATTGAGCGAGT	qRT-PCR for *metR* (SO_0817)
qRT-SO_2906-F	AGCTATTCCGGATGCAGCTA	qRT-PCR for SO_2906
qRT-SO_2906-R	CCACTACGGCTATGGATGGT	qRT-PCR for SO_2906
